# 25th Anniversary Article: Organic Field-Effect Transistors: The Path Beyond Amorphous Silicon

**DOI:** 10.1002/adma.201304346

**Published:** 2014-01-20

**Authors:** Henning Sirringhaus

**Affiliations:** Cavendish Laboratory, University of CambridgeCambridge, CB3 OHE, UK

**Keywords:** organic semiconductors, organic field-effect transistors, organic light-emitting diode displays

## Abstract

Over the past 25 years, organic field-effect transistors (OFETs) have witnessed impressive improvements in materials performance by 3–4 orders of magnitude, and many of the key materials discoveries have been published in *Advanced Materials*. This includes some of the most recent demonstrations of organic field-effect transistors with performance that clearly exceeds that of benchmark amorphous silicon-based devices. In this article, state-of-the-art in OFETs are reviewed in light of requirements for demanding future applications, in particular active-matrix addressing for flexible organic light-emitting diode (OLED) displays. An overview is provided over both small molecule and conjugated polymer materials for which field-effect mobilities exceeding > 1 cm^2^ V^–1^ s^–1^ have been reported. Current understanding is also reviewed of their charge transport physics that allows reaching such unexpectedly high mobilities in these weakly van der Waals bonded and structurally comparatively disordered materials with a view towards understanding the potential for further improvement in performance in the future.

## 1. Introduction

Research on organic field-effect transistors over the past 25 years has contributed greatly to the scientific understanding of the fundamental charge transport physics of conjugated polymer and small-molecule organic semiconductors. These weakly van der Waals bonded materials provide unique realizations of systems in which transport is intermediate between conventional low-mobility hopping transport in amorphous glasses[1] and high-mobility band transport in covalently bonded single-crystals and electronic transfer integrals, on-site energetic disorder as well as diagonal and non-diagonal electron phonon coupling strengths may all be of comparable magnitude.[2] Apart from studies that have been focused on understanding the molecular design guidelines, these materials have allowed fundamental studies of, for example, the degree of wavefunction delocalization achievable in van der Waals bonded materials,[3] the coupling between charge transport and structural dynamics,[4,5] studies of transport at two-dimensional charge transfer interfaces,[6] or the role of nuclear tunneling in electron transfer.[7]

The improved physical understanding of transport and of structure-property relationships has supported the development by organic chemists of better organic semiconductor materials for organic field-effect transistor (OFET) applications as well as other devices that rely on charge transport, such as organic solar cells[8] and light-emitting devices.[9] The field-effect mobility (*μ*), which is the main materials-related figure of merit of an OFET, has increased from low values <10^−3^ cm^2^ V^–1^ s^–1^ 25 years ago to values >1–10 cm^2^ V^–1^ s^–1^ that are now exceeding those of benchmark thin-film amorphous silicon devices (0.5–1 cm^2^ V^–1^ s^–1^). Over this time period, *Advanced Materials* has published many of the key materials advances in the field, and the 25^th^ anniversary of the journal provides an appropriate occasion to review the recent developments in the field and assess their significance in terms of future scientific opportunities and applications.

**Figure 1:**
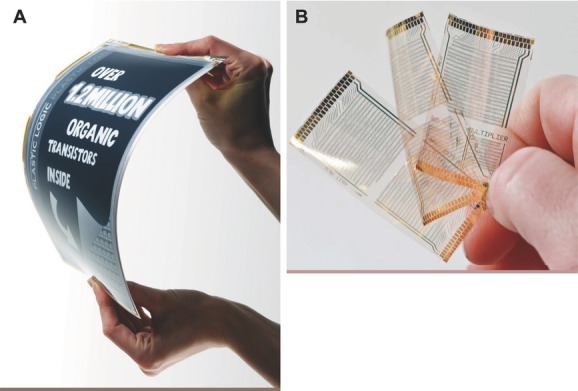
Photographs of A) flexible electrophoretic ink display driven by an active-matrix of 1.2 million OFETs (source: Plastic Logic); B) plastic foils comprising 8-bit microprocessors with 3381 OFETs each. Reproduced with permission.[122] Copyright 2012, IEEE.

The improvements in materials performance have facilitated impressive application demonstrations, including high-resolution, flexible displays based on electrophoretic ink, or “e-paper”, and integrated circuits, such as an 8-bit microcontroller (**Figure** 1).[10] At the same time, significant advances have been made in industrial manufacturing technology. Companies such as Plastic Logic have fully industrialized OFET technology and demonstrated that amorphous silicon performance and LCD industry typical yield and reliability levels can be achieved with OFETs in a full manufacturing environment. OFET-enabled flexible displays are currently finding their way into a wave of first generation applications. With the transistor performance that is readily available today a wide range of applications can already be addressed, in particular, e-paper displays, simple circuits,[10] and chemical and biological sensors.[11] However, stimulated by the recent advances in materials performance and manufacturing technology, the question arises whether OFET technology may also be able to address a wider range of demanding, performance-critical applications beyond e-paper and simple sensors and circuits. A representative and particularly important example is full-color, video, flexible OLED displays. Small OLED displays on conventional glass substrates for mobile phone and PDA applications are rapidly growing and are displacing LCD screens in the small display sector. They may soon enter the mid- to large-size display market for tablets and TVs. The backplane performance requirements for OLED displays are considered to be too demanding for amorphous silicon (a-Si) thin film transistor (TFT) technology, and most of these displays are manufactured using polycrystalline silicon TFTs in spite of the uniformity challenges in manufacturing associated with this technology. Oxide TFTs[12] are also being developed for this application and may soon enter mass production. Both technologies offer field-effect mobilities >20–50 cm^2^ V^–1^ s^–1^, significantly higher than a-Si. Several of the leading display companies have expressed their intention to introduce flexible OLED displays in the near future, which will be lighter and more robust than glass-based displays and will allow novel display applications with new form factors. In principle, OFET technology could be an ideal backplane for this application because of the close materials compatibility between OLEDs and OFETs and their excellent mechanical properties, which might ultimately even allow foldable displays that would require tight bending on flexible substrates to very small radius of curvature on the order of 100 μm.[13] For this, the mechanical properties of OFETs are potentially superior to silicon or oxide based TFTs. The integration of OFETs with OLEDs was demonstrated early,[14] and several groups have realized prototype OFET-driven OLED displays.[10,15,16] However, a commonly held view is that the performance, uniformity and operational stability that is achievable with OFETs is insufficient to realize a backplane that achieves the same display performance and lifetime than a display addressed with oxide or poly-Si TFTs. Part of the aim of this review is to critically assess this view in the light of the recent significant improvements in OFET materials and device performance.

This review is structured as follows. In chapter 2, I discuss materials-related performance requirements for active matrix addressing of current-driven OLED displays. Chapter 3 presents a brief review of recent small-molecule and conjugated polymer semiconductors for which mobilities exceeding 1 cm^2^ V^–1^ s^–1^ have been reported. In chapter 4, I include a brief discussion of potential issues when extracting mobility values from the measured device characteristics that need to be considered to obtain robust mobility values. In chapter 5, I discuss the current understanding of the charge transport physics of some of these materials with a view towards understanding the scope for further improvements in performance in the near future. The review concludes with an outlook for these advances in materials performance to open up new scientific opportunities and to enable addressing the requirements of performance-demanding, next-generation applications, including flexible OLED displays.

### 2. Transistor Performance and Stability Requirements for OLED Active Matrix Addressing

In contrast to voltage-driven liquid crystal or e-paper displays, which merely require the application of a controlled voltage to each pixel of the display, OLEDs are current-driven: the amount of light emitted by a pixel is controlled by the current passing through the OLED. In an active matrix OLED display the current needs to be applied continuously, i.e., not just when the pixels in a particular column are updated but during the entire frame time while all of the other rows of the display are being addressed. A particular pixel may need to be driven continuously for several hours while the display is being used. This translates into TFT performance, stability, and uniformity requirements that are significantly more demanding than those of voltage-driven displays.

**Figure 2:**
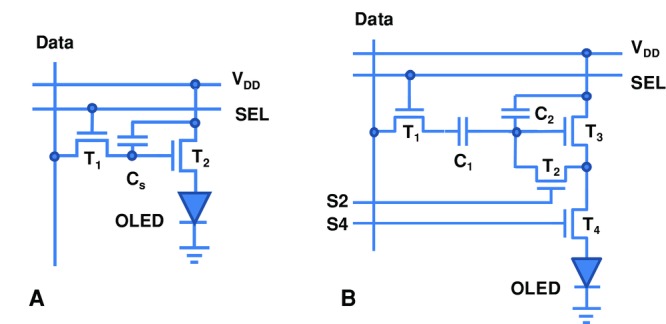
Pixel circuits for active matrix OLED addressing with a p-type OFET: A) simple 2T1C architecture; B) example of voltage compensation circuit comprising 4 pixel TFTs.

The simplest, so-called 2T1C pixel architecture comprises two pixel transistors T_1_ and T_2_ and a storage capacitor C_s_ integrated with the OLED (**Figure** 2A). A particular pixel is selected for updating by switching on the transistor T_1_ and writing a controlled voltage onto the storage capacitor, C_s_. This voltage in turn switches on the gate of the drive transistor T_2_ and controls a programmed drive current to the OLED supplied by T_2_. This current is maintained when T_1_ is switched off again to address the next row of the display. For an OLED with a standard bottom-contact anode and a p-type (organic) transistor the OLED anode is connected to the drain of the transistor and the voltage *V*_DD_ is typically selected such that T_2_ operates in saturation, so changes in the voltage drop across the OLED do not directly lead to changes in the intensity of the emitted light. To achieve the same beneficial effect with an n-type (oxide) transistor the OLED would have to be constructed in an inverted configuration with the cathode on the bottom. This simple circuit allows the basic active matrix addressing of a current-driven display. It requires transistor T_2_ to be able to supply a sufficient current density to the OLED so as to reach the required front-of-screen display brightness *B*, which may be on the order of 350 Cd m^–^^2^ for a mobile application. For an OLED with an efficiency *η* = 6 Cd/A, which is typical for state-of-the-art blue OLEDs, a geometric channel width to channel length (*W/L*) ratio of the TFT, which needs to be compatible with the display resolution and patterning design rules, translates into a minimum requirement for the mobility:

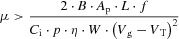
(1)where *C*_i_ is the gate dielectric capacitance, *V*_g_ is the applied gate voltage, *V*_T_ is the threshold voltage of the TFT, *A*_p_ is the pixel area (127 μm × 127 μm for a 200 ppi display), *p* is the transmission of the circular polarizer commonly used to reduce glare (assumed 50%), and *f* is the fraction of (blue) light that needs to be mixed in to make white light (depends on the colour coordinates; assumed to be 20%). These conditions correspond to a typical OLED current density for the (blue) subpixel of 10 mA cm^–^^2^ (assuming an aperture ratio of 50%) or an absolute subpixel current on the order of 250 nA. For *W/L* = 2 and *V*_g_*–V*_T_ = 5V, this imposes a minimum mobility requirement of *μ* > 1.5 cm^2^ V^–1^ s^–1^.

This drive-current imposed mobility requirement appears feasible for a state-of-the art OFET; however, it does not constitute the most stringent mobility requirement for this application. This is because the simple 2T1C architecture has a number of shortcomings. If the threshold voltage, *V*_T_, of T_2_ exhibits manufacturing related non-uniformities across the display, this manifests itself directly in spatial non-uniformities of the emitted light intensity. Similarly, temperature variations that change the TFT characteristics directly lead to changes in the displayed image. Furthermore, if the *V*_T_ of any pixel increases owing to the operational stress experienced during the continued operation of the display, this directly results in a gradual reduction of the light output from the stressed pixel for a given programmed gate voltage. To overcome such degradation temporal changes and spatial non-uniformities in device characteristics need to be measured, and a dedicated compensation circuit on each pixel requiring typically four or more TFTs per pixel and a more complex drive scheme is needed to retain the programmed current through the OLED. A variety of such circuits have been proposed; they can be categorized into voltage and current programming schemes.[17] Current programming schemes use a current mirror topology to self-adjust the gate-source voltage of the drive TFT. In voltage programming, the threshold voltage of the drive TFT is generated across its source-gate junction and added to the programmed pixel voltage (Figure 2B). The need to operate such compensation circuits at a sufficient speed—i.e., during the short available pixel addressing time—tends to impose a more stringent mobility requirement than the OLED drive current requirement of Equation 1. It has been estimated that a mobility of 5–10 cm^2^ V^–1^ s^–1^ provides a more realistic minimum requirement for a typical voltage compensation circuit to operate at a sufficient speed.[18] However, the detailed mobility requirement is dependent on factors such as the spatial uniformity of the transistor characteristics across the display and the operational stability.

Compensation circuits typically work only as long as the gate-source voltage of the drive TFT does not have to be increased to such a high value that it stops the TFT operating in the saturation regime for given applied *V*_DD_ and OLED voltage drop *V*_OLED_. If during an applied constant current stress the TFT threshold voltage degrades according to a power law[19]

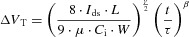
(2)with parameters *γ*, *τ*, and *β* characterizing how the degradation depends on applied current/gate voltage and stress time, it is possible to predict the “lifetime” *t_max_* of the pixel

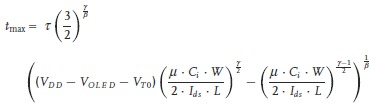
(3)where *V*_T0_ is the initial threshold voltage of the TFT.[18]

Detailed assessment of the required drive TFT stability will in general require full modeling taking into account the pixel compensation circuit and the drive scheme. However, the requirement expressed by Equation 3 provides a basic but useful criterion for materials scientists to assess new OFET materials or device architectures in terms of their suitability for OLED applications. To apply it, constant current stress measurements—in which the gate-source voltage is continuously adjusted to keep the source-drain current constant at a value that would supply sufficient current to an OLED to reach maximum brightness (Equation 1)—need to be performed to extract the parameters *γ*, *τ*, and *β*. Unfortunately, at present there are almost no such measurements reported for OFETs in the literature. Constant voltage stress measurements are more commonly performed,[20,21] but they are also less useful in an OLED context, as they involve monitoring the current decay at a fixed gate voltage and therefore tend to stress the TFT less than a constant current measurement. Such measurements should be reported for new high-performance OFET materials and devices. As a guideline, to reach an acceptable display brightness for a (blue) OLED pixel in a 200 ppi display the driving TFT, which will typically have to be limited to a *W/L* ratio of 2–5 in order not to occupy a too large fraction of the pixel has to supply a current on the order of 250 nA continuously. A useful stress protocol may therefore be to monitor the gate voltage required to maintain a constant current on the order of 250 nA as a function of time in a device with *W/L* = 5 (or proportionally higher current if a larger *W/L* is selected). The measurement may be performed over a practical stress period of one or several days, at elevated temperature, if necessary, to accelerate any degradation effects. The recovery kinetics of any threshold voltage shifts should also be investigated. A useful benchmark is the stability that can be achieved with state-of-the-art oxide TFTs, which typically exhibit threshold voltage shifts of less than 1V during such a current stress measurement over a period of 100 h at room temperature.[22]

### 3. Recent Advances in Organic Semiconductor Materials for OFETs

In this section, I review state-of-the art organic semiconductor materials that have allowed to realize OFETs with high mobilities >1 cm^2^ V^–1^ s^–1^, which may approach the mobility requirements to address OLEDs. I first discuss small-molecule-based materials, which have traditionally exhibited higher mobility values than conjugated polymers. In the second section, I turn to advances in low bandgap donor–acceptor (D–A), conjugated polymers that have recently reached performance levels comparable to those of small molecules. We limit our discussion to solution-processable materials, which now offer comparable performance, but potential processing advantages to vacuum sublimed materials, and to p-type devices, which tend to exhibit better environmental and operational stability. For a more complete discussion of OFET materials see the following excellent recent review articles.[23–26]

In small-molecule systems, the basic molecular design criteria for achieving high mobilities are comparatively well understood. The field effect mobility of an organic FET depends sensitively on the molecular structure and the intermolecular packing, which determine the reorganisation energy and the transfer integral that govern charge transport.[27] For high-mobility materials, the reorganization energy should be small, and the transfer integral should be large. In addition, static structural and energetic disorder encountered by the charges moving along the interface should be minimized by material purification and suitable processing and interface control. Although the occurrence of such defects may in many cases be an extrinsic, processing, or purification-related effect, there may also be important molecular design rules that affect the propensity of certain molecular structures towards formation of structural defects. These are generally less well understood. Finally, one should carefully consider the role of thermal lattice fluctuations that can lead to dynamic modulations of the intermolecular packing and the associated charge transfer integrals on the same timescale as carrier motion. These require charge carriers to remain localized on particular lattice sites until a favourable molecular configuration for charge transfer occurs dynamically.[28–30]

**Figure 3:**
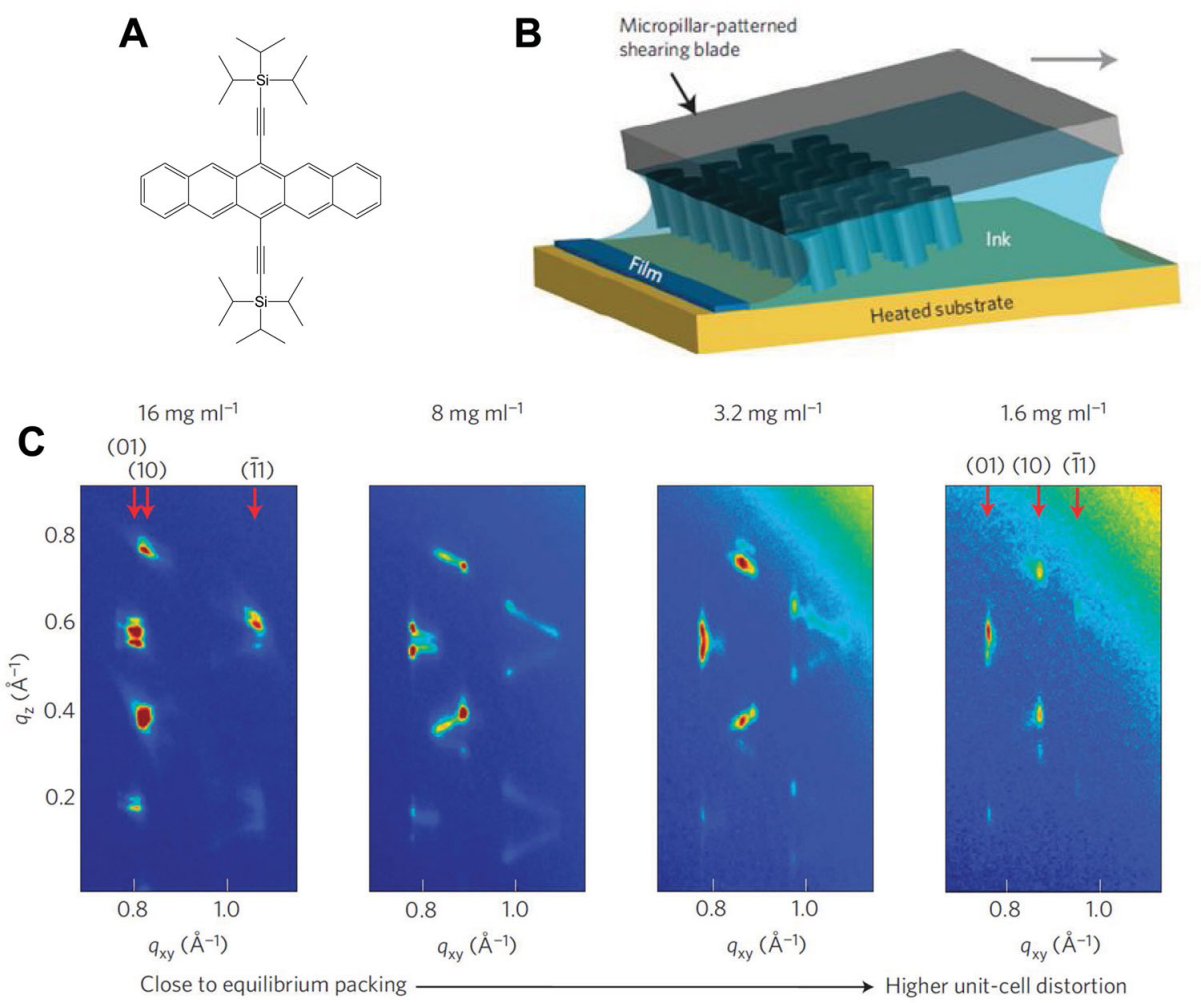
Growth of strained TIPS-pentacene polymorphs by blade coating technique with micropillar improved fluid flow: A) molecular structure of TIPS-P; B) schematic diagram of coating process; C) wide-angle X-ray scattering (WAXS) images of different TIPS-P polymorphs obtained by coating films from different solution concentration at a shearing speed of 0.8 mm s^–1^. Reproduced with permission.[39] Copyright 2013, Macmillan Publishers Ltd.

Many of the highest-performing small molecules are based on acenes and fused heteroacene materials. Significant efforts continue into a class of solution-processible acenes based on the excellent solubility characteristics of the trialkylsilylethynyl group, such as triisopropyl-silylethynyl (TIPS), substituted on the central 6- and 13-position of a pentacene core, which was first developed by Anthony et al.[31] Spin-coated TIPS-pentacene (TIPS-P) films exhibit a high degree of crystallinity with a two-dimensional, brick-wall, cofacial π−π-stacking motif in the plane of the films with the side chains oriented near normal to the substrate surface. Mobilities exceeding 1 cm^2^ V^–1^ s^–1^ were obtained early in films of neat TIPS-P.[32] Anthony's group has investigated the structure–property relationships of a wide range of solution-processible, trialkylsilylethynyl substituted acenes and has studied the correlation between the crystal packing motif and carrier mobility and found that 1D, π−π stacking tends to result in lower mobility than 2D, brick-wall packing.[33] Various heteroacene derivatives, such as triethylsilylethynyl anthradithiophene (TESADT)[34] or difluorinated (di-F) TESADT[35] exhibit similar levels of performance to TIPS-P. Control of device to device uniformity over the area of the substrate is a common challenge in these solution-processible small molecules that have a strong tendency to crystallize. Several groups have reported improved device-to-device uniformity when blending the small molecule with a polymer binder.[36] In blend films of TIPS-P and diF-TESADT, respectively, in which the small molecule tends to segregate to the surface and interface of the films Hamilton et al.[37] reported mobilities of 1 cm^2^ V^–1^ s^–1^ and 2.4 cm^2^ V^–1^ s^–1^ respectively. The choice of binder polymer exerts a significant influence on the crystallinity of the small molecule and with an optimized binder polymer diF-TESADT devices were more recently improved to mobilitiy values of 4–5 cm^2^ V^–1^s^–1^.[38] Recently, Giri and co-workers[39,40] reported that by using a blade-coating technique with a controlled shear strain applied during growth, it is possible to induce a remarkable range of strained, non-equilibrium polymorphs of TIPS-P and achieve mobilities up to 11 cm^2^ V^–1^ s^–1^ under optimum growth conditions (**Figure** 3).

**Figure 4:**
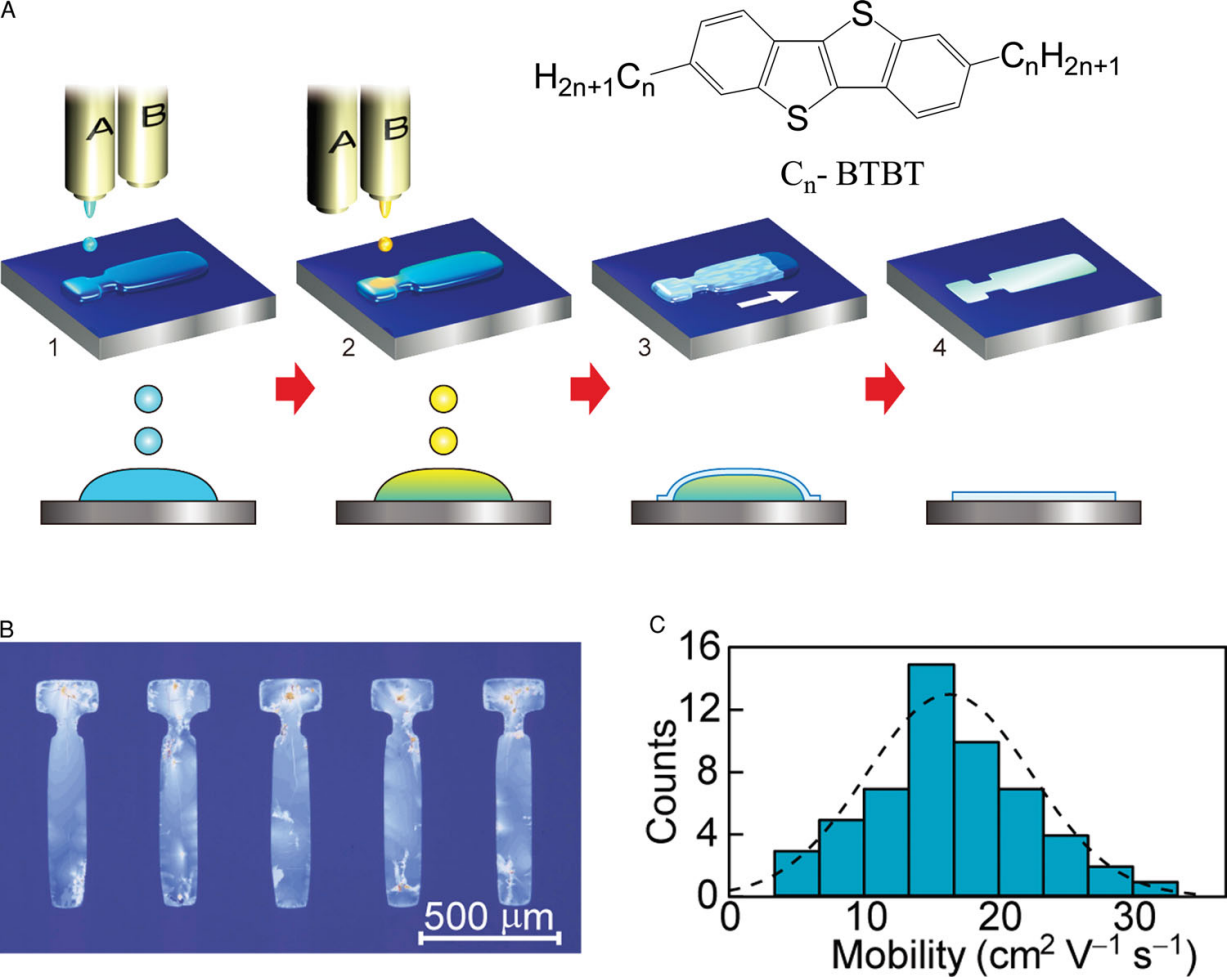
Double-shot inkjet printing process of C_*n*_-BTBT films: A) schematic diagram of process printing subsequently ink droplets of an antisolvent (blue) and then droplets of the ink comprising the organic semiconductor (yellow) onto a substrate with a surface energy pattern confining the ink droplets; the inset shows the molecular structure of C_*n*_-BTBT; B) polarized optical micrograph of printed C_8_-BTBT films; C) histogram of field-effect mobilities across 54 top-gate, top-contact transistors. Reproduced with permission.[44] Copyright 2008, Macmillan Publishers Ltd.

Takimiya's group invented a class of end-substituted phenylene-thiophene, selenophene or thiazine fused ring systems that have reached some of the highest device performance in thin film OFETs to date.[41,42] Spin-coated films of di-alkyl end-substituted derivatives of benzothienobenzothiophene C_*n*_-BTBT (*n* = 11–13) with a highly ordered, crystalline microstructure and alternating layers of aliphatic side chains and conjugated layers parallel to the substrate exhibit high mobilities of 1–3 cm^2^ V^–1^ s^–1^. C_*n*_-BTBT with longer sides chains have slightly higher mobility than C_*n*_-BTBT with shorter side chains, because the stronger hydrophobic interaction induced by the longer side chains tends to enhance the conjugated molecular overlap. By drop-casting onto an inclined substrate the film morphology for C_8_-BTBT could be optimized and bottom-gate FETs with mobilities of 5 cm^2^ V^–1^ s^–1^ were reported.[43] Minemawari et al.[44] realized some of the highest performance solution-processed OFETs reported to date with average and maximum mobilities of 16.4 cm^2^ V^–1^ and 31.3 cm^2^ V^–1^ s^–1^, respectively, by using a two-shot inkjet printing technique during which a thin film of C_8_-BTBT is made to crystallize on the liquid surface of an antisolvent droplet. By confining the printed droplet within a specially designed surface energy patterned region that includes a nucleation zone connected to the active device region by a narrow neck growth of near single-crystalline domains can be obtained in the active region (**Figure** 4). C_*n*_-BTBT derivatives exhibit low melting points around 120 °C[45] and also have a relatively deep ionization potential which requires careful device processing and contact optimization.[46] Di-alkylated C_*n*_-DNTT has a higher melting point,[47] but it exhibits only moderate solubility, which is too low for forming films by spin coating. However, it shows excellent device performance with mobilities up to 12 cm^2^ V^–1^ s^–1^ when deposited by a drop-casting technique onto an inclined substrate with an edge pinned contact line.[48,49] The devices exhibit band-like transport characteristics with an ideal Hall signature and mobility increasing with decreasing temperature.[50] A range of other alkylated phenylene-thiophene oligomers have also yielded high performance, including solution-grown microcrystals of alkylated dithienothiophene with mobilities up to 10.2 cm^2^ V^–1^ s^–1^[51] and vacuum-sublimed films of monoalkyl-functionalised BTBT (17.2 cm^2^ V^–1^ s^–1^)[52] and bisbenzothienonaphtalene (15 cm^2^ V^–1^ s^–1^).[53]

Apart from acene and fused thiophene-phenylenes there is a range of other molecular design motifs that have been found to be promising. Examples include titanylphtalocyanine (TiOPc), which has a non-planar, square pyramid shape, and exhibits mobilities of up to 3.6 cm^2^ V^–1^ s^–1^ in vacuum grown films on OTS-modified SiO_2_.[54] In solution-grown crystals of dithiophene- tetrathiafulvalene (TTF) field-effect mobilities of 3.6 cm^2^ V^–1^ s^–1^ were observed.[55] Solution grown crystals of hexamethylene-TTF (HMTTF) contacted with TTF-TCNQ source-drain electrodes mobilities of 10 cm^2^ V^–1^ s^–1^ were reported. The mole­cules adopt a 2D, cofacial, brick-wall π−π stacking motif.[56]

We now shift our discussion to high mobility conjugated polymers. Although it is possible to achieve reasonably high mobilities of 10^−3^–10^−2^ cm^2^ V^–1^ s^–1^ using fully amorphous materials as long as the polymer is designed to minimize energetic disorder,[57–59] the most successful and widely explored design motif for high-mobility conjugated polymers has traditionally been that of semicrystalline lamellar microstructures with edge-on polymer orientation similar to that found in P3HT[60] or poly(3,3′-dialkyl-quaterthiophene) (PQT).[61] Through the formation of alternating layers of conjugated backbones separated by layers of flexible side chains parallel to the substrate plane efficient charge transport in the plane of the film along the active interface of the OFET is facilitated, because the motion of charges is not impeded by the presence of the flexible, insulating side chains. The most beautiful realization of this highly semicrystalline structure occurs in poly(2,5-bis(3-alkylthiophen-2-yl)thieno(3,2-b)thiophene) (PBTTT), in which mobilities up to 1.1 cm^2^ V^–1^ s^–1^ have been observed.[62,63] The lower density of side chains in thiophene–thienothiophene copolymers, such as PBTTT, not only slightly increases the ionization potential and improves the stability, but allows for better side-chain interdigitation and more highly crystalline structures. This work has recently been reviewed by McCulloch et al.[64]

At present, there is significant research focus on investigating the charge transport properties of donor-acceptor copolymers. These have significantly more complex backbone structures and larger conjugated units than P3HT or PBTTT. They are usually regular copolymers with alternating electron deficient and electron rich units along the polymer backbone, that exhibit a relatively low band gap associated with an intramolecular charge transfer transition between the electron rich and electron deficient units. This makes them very attractive for solar cell applications. In the context of FETs, although there is not necessarily a direct link between band gap/energy levels and mobility, the low band gap facilitates the selection of suitable electron/hole injecting contacts for efficient charge injection/extraction and avoidance of charge trapping in states induced by atmospheric species or chemical impurities in the semiconductor or gate dielectric layer at the interface. Furthermore, it is often argued that potentially an attractive intermolecular interaction between donor and acceptor units stacked on top of each other could lead to small π−π stacking distance and efficient intermolecular charge transfer, although direct experimental evidence for the presence of such donor-acceptor stacks is needed in each system to ascertain this. Unfortunately, this is experimentally hard to obtain from structural measurements such as grazing incidence X-ray diffraction (GIXRD) and requires sophisticated structural probes such as 2D-NMR.[65]

Two of the first examples of high mobility donor-acceptor polymers were a copolymer of naphthalenediimide and bithiophene (PNDI2OD-T2), for which n-type, electron mobilities of 0.8 cm^2^ V^–1^ s^–1^ were observed,[66] and a copolymer of cyclopentadithiophene and benzothiadiazole (CDT-BTZ)[67] that exhibits mobilities up to 3.5 cm^2^ V^–1^ s^–1^ in dip-coated thin films on HMDS modified SiO_2_ gate dielectric and up to 5.5 cm^2^ V^–1^ s^–1^ in single fibres grown by a solvent vapour enhanced drop casting technique.[65,68] Both polymers were first thought to be amorphous, but were later found to be semi-crystalline. NDI-T2 adopts an unusual face-on π-stacking[69] while CDT-BTZ adopts a semicrystalline, edge-on, lamellar structure with close π−π stacking distance of 3.7–3.8 Å.

The class of D–A polymers that has possibly received the most attention to date are low-bandgap copolymers based on the electron-deficient unit of diketopyrrolopyrrole (DPP). DPP is a highly photostable, red synthetic pigment that is being used widely in building or automobile coatings. The first report was a copolymer based on DPP and thiophene, which not only exhibited good hole mobilities of 0.1 cm^2^ V^–1^ s^–1^, but also electron mobilities of the same magnitude and could be operated as an ambipolar, infrared emitting light-emitting transistor.[70] By copolymerizing electron-deficient, di-aryl-DPP units with a wide range of electron donor units, more than eighty low-bandgap copolymers have since been synthesized and evaluated in FET devices. In a DPP copolymer with thienothiophene-based acceptor units, the first realization of an ambipolar top-gate polymer FET in which both the electron and the hole mobility exceed 1 cm^2^ V^–1^ s^–1^ was achieved.[71,72] In the same polymer using a bottom-gate architecture with self-assembled monolayer modified SiO_2_ gate dielectric, Ong's group[73] has claimed recently mobilities of up to 10 cm^2^ V^–1^ s^–1^ as a result of increased molecular weight. Similarly, high performance has also been reported in the same device structure using DPP copolymers that comprise vinylene linkages[74,75] For a recent, complete review over the evaluated DPP materials, see the excellent review by Nielsen et al.[24]

Two other classes of donor acceptor copolymers that appear very promising are isoindigo (IID)- and indacenodithiophene (IDT)-based copolymers. In an IID copolymer with bithiophene mobilities approaching 1 cm^2^ V^–1^ s^–1^[76,77] were first reported. More recently higher mobilities of 3.6 cm^2^ V^–1^ s^–1^ were achieved through optimization of the distance of the branching point of the alkyl side chains from the conjugated backbone[78] and through use of siloxane terminated side chains.[79] In an indacenodithiophene-based copolymer with benzothiadiazole (IDT-BT), mobilities of 1.2 cm^2^ V^–1^ s^–1^ were first reported by McCulloch's group[80] and more recently values up to 3.6 cm^2^ V^–1^ s^–1^.[81]

**Figure 5:**
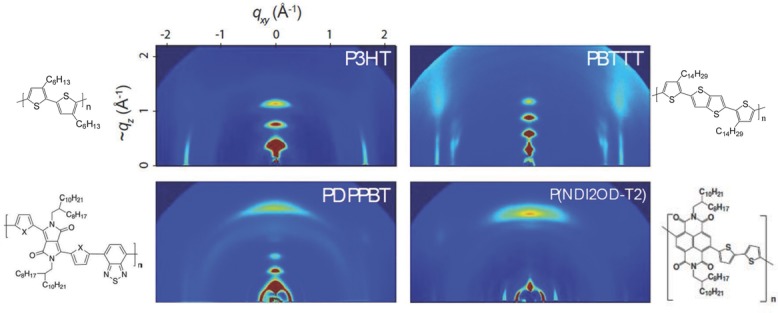
Wide-angle X-ray scattering images of different high-mobility conjugated polymers. The semicrystalline, thiophene-based polymers P3HT and PBTTT are semicrystalline and exhibit well-defined, relatively sharp diffraction spots including several orders of diffraction and a higher anisotropic orientation with respect to the substrate plane, while the donor-accept copolymers, PDPPBT and PNDI2OD-T2 exhibit a still semicrystalline, but less-ordered microstructure with fewer and wider diffraction spots. Reproduced with permission.[98]_Copyright 2013, Macmillan Publishers Ltd._

The structure–property relationships of these high-mobility conjugated polymers and a microscopic understanding of the origin of such unexpectedly high mobilities are not well understood at present. What seems remarkable is that these polymers do not appear to follow the design guidelines for high-mobility polymers that were established by early work on P3HT, i.e., the need for a semicrystalline, edge-on lamellar microstructure allowing efficient in-plane transport both along the polymer backbone and the direction of π−π stacking. Many of the new D–A copolymers perform better than semicrystalline P3HT or PBTTT in spite of exhibiting significantly less pronounced crystalline order with fewer and wider diffraction spots in GIXRD and in some cases even a face-on, preferential orientation (**Figure** 5). The exceptionally high mobilities are clearly not an extension of the trend to higher degrees of semicrystalline order that was established when moving from P3HT to PBTTT. In Section 6, we provide a discussion of the emerging understanding of their charge transport physics. We conclude this materials review section by stating that there is now a wide range of solution-processible organic semiconductors for which performance levels beyond that of amorphous silicon have been demonstrated. A few materials are entering into a mobility regime above 10 cm^2^ V^–1^ s^–1^, where demanding applications such as OLED active matrix addressing may become feasible.

### 4. Extraction of Field-Effect Mobilities

Before continuing, I feel compelled to insert a brief discussion of the experimental methods that have been used to extract mobilties. As organic TFTs are being seriously assessed for mobility critical applications, such as current driven flexible OLED displays, it is becoming increasingly important that the mobility values that are being reported are not overestimated and reflect the true current driving capability of the device. This is an important issue because some of the high mobility materials discussed above exhibit non-idealities in their transfer and output characteristics, which make it difficult to apply standard mobility extraction methods. For a device with “near”-ideal transfer characteristics the current *I*_d_ varies with gate voltage *V*_g_ for a given source-drain voltage *V*_sd_ in the linear and saturation regimes, respectively, according to

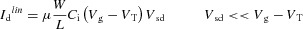
(4)

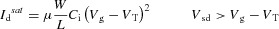
(5)

**Figure 6:**
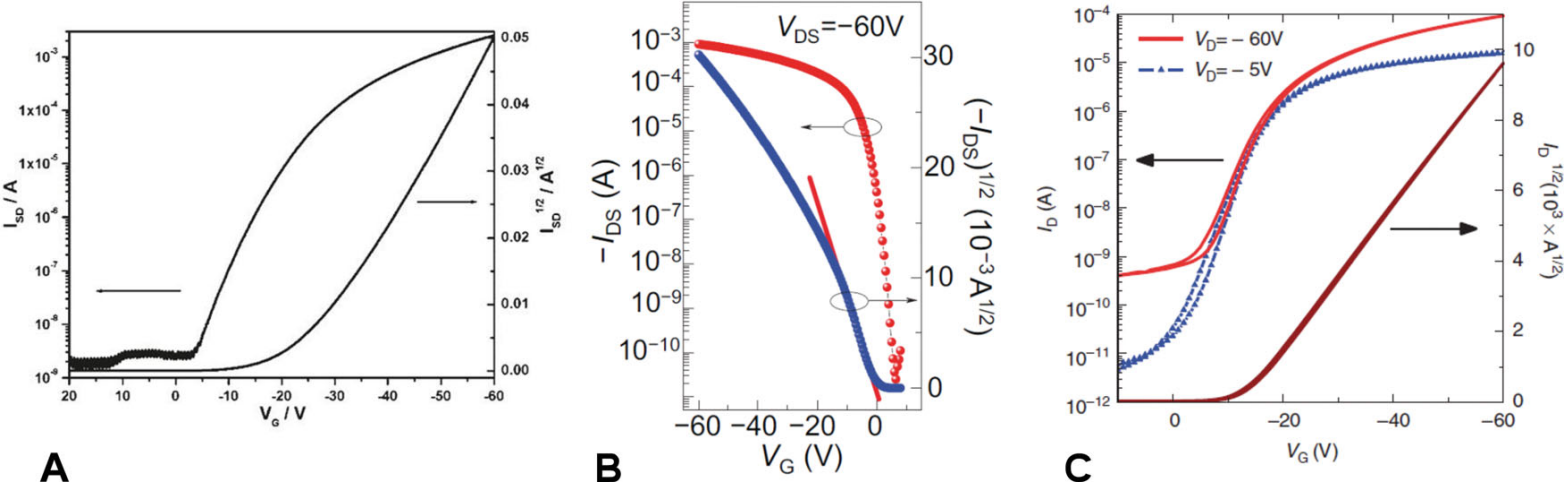
Illustration of methods of mobility extraction from high-mobility conjugated polymer OFETs which exhibit non-ideal transfer characteristics: (A) CDT-BTZ bottom-gate, top-contact FET. Reproduced with permission.[65] Copyright 2011, American Chemical Society. B) DPP-T-TT bottom-gate, top-contact FET. Reproduced with permission.[73] Copyright 2012, Macmillan Publishers Ltd. C) IDTBT top-gate, bottom-contact FET. Reproduced with permission.[81] Copyright 2013, Macmillan Publishers Ltd.

The mobility can reliably be extracted from a linear fit of the gate voltage dependence of the current in the linear regime or the square root of the current in the saturation regime. However, questions arise when the device exhibits deviations from this ideal behavior. Three of the most common issues encountered are:

- In some materials the device characteristics exhibit hysteresis with the current being higher on the forward sweep towards larger ON voltages than on the reverse sweep. This is usually due to the current in the FET degrading on the timescale of the electrical measurement, and this instability reflects dispersive transport and charge trapping in the organic semiconductor or at the interface. If the sweep rate is increased to mask this instability, or if the reverse transfer characteristics are used to extract mobility, this can lead to overestimated mobility values that do not reflect an equilibrium charge carrier concentration and continuous current carrying capability of the OFET.[82]
- The slope of the square root of the current versus gate voltage in the saturated transfer characteristics increases with increasing magnitude of the gate voltage (**Figure** 6A). This behavior, which is commonly observed in semicrystalline polymers, such as P3HT and PBTTT, has also been observed in several, high mobility D–A copolymers.[65,72] It is typically associated with the presence of localized, “low-mobility” states in the tails of the density of states, which need to be filled before charge carriers can access parts of the density of states with more delocalized, “high-mobility” states. In this case, mobility extracted from a linear fit in a limited gate voltage range at high gate voltages may be interpreted as a genuine transport parameter that may potentially be realized across the full gate voltage range if disorder in the bulk or at the interface could be minimized. It may, however, also be a manifestation of contact resistance: for example, a contact resistance that decreases more rapidly with increasing gate voltage than the channel resistance. In the latter case, mobility extracted from a limited gate voltage range at high gate voltages could provide an overestimate and may not be realizable in an optimized device with lower contact resistance.
- The slope of the square root of the current versus gate voltage in the saturated transfer characteristics is high at small gate voltage but decreases with increasing magnitude of the gate voltage (Figure 6B). This has been observed for several high-performing D–A copolymers in bottom gate FETs with SAM modified SiO_2_ gate dielectric.[73–75,78,83] A possible explanation, which has been proposed for phenomenologically similar behavior observed in rubrene single crystal devices,[84] may be that at lower gate voltage the accumulation layer is not as tightly confined to the interface and extends further into the bulk than at high gate voltages where charges are more tightly attracted towards the interface. If the degree of disorder is lower in the bulk than at the interface, this might result in the observed behavior. Alternatively, such behavior may be attributed to contact resistance effects: the current becomes more contact-limited as the interfacial accumulation layer becomes more conducting at high gate voltages.[85] Yet another explanation might be that at higher gate field, some charges from the accumulation layer are injected into the gate dielectric or the interface modification layer and become trapped. If these situations apply—this will need to be established in each case through suitable control experiments—a mobility value extracted by linear fitting of only the low voltage region of the square root of the saturated transfer characteristics may reflect a meaningful materials or transport parameter, although it does of course not reflect fairly the practical current drive capability of the device as the high mobility cannot be maintained at high voltages. This would be more fairly represented by quoting as well a more conservative mobility estimate extracted from the (lower) slope in the high gate voltage regime. In any case, it should be avoided to extract mobilities from very narrow, low gate voltage regions of only a few volts, since such voltage regions may still be in the subthreshold regime where Equations 4 and 5 do not apply.

These issues are important to take into account and for particular systems require detailed device physics studies in order to identify the origin of the non-ideality. In general, conservative mobility estimates are preferable. However, we would like to emphasize that these issues of mobility extraction do not question the general conclusion of section 3 that there is now a broad range of organic semiconductors that significantly exceed mobilities of amorphous silicon. Many of the recently reported high-mobility materials exhibit near ideal transfer characteristics[65,81,86] (Figure 6C) and, even in those materials that do not, further materials and device optimization may result in more ideal behaviour.

### 5. Transport Physics of High Mobility D–A Copolymers

**Figure 7:**
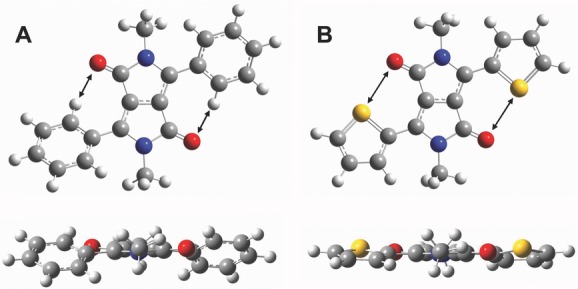
Theoretical simulations of conformations of diphenyl-DPP (A) and dithienyl-DPP (B) in front view (top) and side view (bottom) illustrating the increased torsional backbone twist (27° dihedral angle) in diphenyl-DPP relative to dithienyl-DPP (12° dihedral angle). Reproduced with permission.[24]

**Figure 8:**
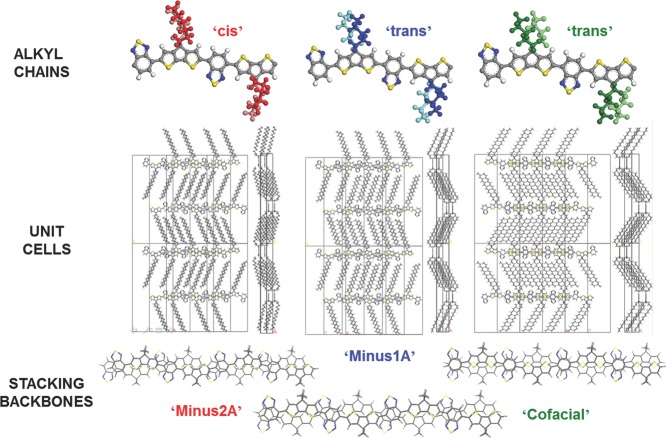
Three polymorphs of CDT-BTZ identified from molecular dynamics simulations and suggested on the basis of NMR data. ‘Cofacial’ corresponds to a perfect matching between the donor and acceptor units belonging to neighbouring chains, while ‘Minus1A’ (‘Minus2A’) has conjugated backbones shifted longitudinally by 1 (2) Å, with respect to ‘Cofacial’. The Minus2A polymorph agrees best with experimental GIXRD and 2D-NMR data. Reproduced with permission.[89]

Although in small-molecule organic semiconductors, there appear to be clear design guidelines for high-mobility materials, which include a high degree of crystallinity with close π−π stacking, extended π−π overlap in ideally more than one spatial direction, reduced propensity to formation of static lattice defects and suppression of thermal lattice fluctuations, the high charge carrier mobilities that have recently been observed in donor-acceptor polymers have come as a surprise. As stated above, the exceptionally high mobilities in these materials can clearly not be understood as an extension of the trend to higher and higher degrees of semicrystalline order that was established when moving from P3HT to PBTTT (5Figure ). In this section, I aim to discuss the emerging understanding of the charge transport physics of high-mobility donor–acceptor copolymers. I begin by describing some of the empirical molecular design requirements that appear to be important for achieving high mobilities in these apparently comparatively poorly ordered systems:

- One factor that appears to be important is a coplanar backbone conformation with minimum backbone torsion and steric hindrance between the donor and acceptor conjugated units. Evidence for this stems, for example, from comparison of di-aryl-based DPP copolymers with different aryl groups flanking the DPP core. Polymers in which a five-membered thiophene ring is adjacent to the DPP unit exhibit significantly higher performance than polymers comprising a phenylene ring adjacent to the DPP. Di-thienyl-DPP polymers can adopt a more planar backbone conformation than di-phenyl-DPPs owing to reduced steric hindrance between the DPP and thiophene units (**Figure 7**). A planar backbone conformation minimizes the scope for structural disorder, enables chains to pack closely with small π−π stacking distances of 3.6-3.8Å and tends to reduce the torsional contribution to the reorganization energy for electron transfer. Also, in IDT-BT and CDT-BTZ, a highly planar backbone conformation is likely to be an important motif. There is less steric hindrance between the IDT/CDT donor unit and the BT acceptor unit than, for example, between the fluorene and the BT unit in structurally closely related F8BT, which only exhibits electron and hole mobilities on the order of 10^−3^–10^−2^ cm^2^ V^–1^ s^–1^. One important exception to this rule is PNDI2OD-T2, in which a significant torsion angle is predicted between the NDI donor unit and the T2 acceptor unit.[87] Although this non-planar backbone confirmation clearly does not prevent achievement of exceptionally high electron mobilities in this polymer it may be the reason that many other NDI-based copolymers with different acceptor units are unable to adopt a similarly favorably microstructure and exhibit comparatively lower device performance.[88] In the DPP class of polymers there appears to be a wider range of acceptor units that lead to high performance than in the NDI family of polymers.
- An important question that has not yet generally been answered is whether attractive, electronic interactions between the donor and acceptor units contribute to achieving a close π−π stacking in these materials. One of the experimental problems to answer this question is that there are only a few experimental techniques that provide direct experimental information about the intermolecular packing in these materials, i.e., whether donor units stack preferentially with acceptor units of the neighbouring chain or with adjacent donor units. This is difficult to predict theoretically as the interchain packing in these polymers is influenced not only by π−π interactions, but also strongly dictated by the need for the side chains to form a low-energy, space-filling structure. Two-dimensional nuclear magnetic resonance (2D-NMR) is one of the few techniques that provides information about the interchain packing. However, these measurements have so far only been performed for very few polymers. In CDT-BTZ a donor-to-donor and acceptor–acceptor stacking motif was observed, which suggests that attractive donor–acceptor interactions may be less relevant for promoting close π−π stacking than first believed.[65] A careful analysis of the GIXRD and 2D-NMR data on this polymer combined with density functional theory molecular modelling identified predominantly donor-on-donor/acceptor-on-acceptor stacking with a small shift along the polymer backbone as the most likely intermolecular packing geometry (**Figure** 8).[89] More materials should clearly be investigated in this way.
- A high degree of in-plane alignment of the polymer backbone facilitating efficient transport along the backbone appears to be crucial. There is convincing evidence that in several of the high-mobility DPP copolymers, the degree of in-plane alignment of the polymer backbone can be exceptionally high, higher than in polymers such as PBTTT.[90] Similarly, IDT-BT exhibits a high degree of order associated with the in-plane alignment of the polymer backbone manifesting itself in in-plane diffraction spots associated with the repeat unit along the polymer backbone up to the third order.[81] This is also consistent with observations in many systems, such as DPP-T-TT[73] and CDT-BTZ,[65] that the mobility increases with molecular weight. Such an increase of mobility with increasing molecular weight at low/intermediate molecular weights is commonly observed in many conjugated polymers, including P3HT. However, it is not expected to extend into a high-molecular-weight regime, setting in typically around degrees of polymerization of 20–50 in which the polymer chains become entangled and the mobility is expected to saturate. In this respect the observation[73] that mobility continues to increase strongly up to very high molecular weights *M*_n_ > 100 kg mol^–1^ may possibly be related to the difficulty of measuring accurately the molecular weight of these copolymers due to their tendency to aggregate in the concentrated solutions used for gel permeation chromatography.[83]
- The question of whether an edge-on as opposed to a face-on orientation of the conjugated backbone units with respect to the substrate plane is as important as it is believed to be in semicrystalline, thiophene-based polymers such as P3HT and PBTTT[64,91] remains unclear. PNDI-T2 was the first high mobility polymer in which clear evidence for a predominantly face-on orientation of the polymer crystallites was found.[92] It was also shown that a rather dramatic change from face-on orientation to edge-on orientation could be induced by high-temperature annealing which did not result in significant differences in mobility,[69] suggesting that the performance may not in fact be very sensitive to whether the crystallites are oriented face-on or edge-on. A few, reasonably high-performance DPP copolymers have also been found to adopt a predominantly face-on orientation[90] or mixed edge-on/face-on orientation.[93] It has been argued that in the face-on orientation, although side chains oriented in plane will undoubtedly constitute difficult hops for charge carriers at the interface, the direction of fast electron transport along the covalently bonded polymer backbone is still predominantly in-plane and the close π−π stacking in the direction perpendicular to the interface will enable polymer layers other than the first monolayer in contact with the gate dielectric to participate in transport. This should provide efficient transport pathways, particularly when different domains with different backbone orientation overlap in the vertical direction as recently observed for PNDI2OD-T2 in high-resolution transmission electron microscopy.[94] On the other hand, the best performing DPP copolymers, for which mobilities in excess of 2–3 cm^2^ V^–1^ s^–1^ have been reported, such as PDVT,[74] DPP-T-TT,[72,73] a thiazolothiazole DPP copolymer[95] and a selenophene-based DPP copolymer with branched siloxane side chains[96] all adopt a predominantly edge-on, lamellar microstructure, which facilitates in-plane transport both along the polymer backbone as well as along the direction of π−π stacking.
- An important aspect of the molecular design of high mobility D–A copolymers relates to the choice of the flexible, solubilizing side chains. The need for the side chains to form low-energy, space-filling structures can largely dictate the self-organisation of the polymer during solution growth, i.e., the attractive van der Waals interactions between the flexible alkyl side chains may exert a more important influence on the interchain packing and backbone conformation than the π−π interactions between the conjugated units. The impact of side chain substitution on the solid state microstructure of conjugated polymers is still not possible to predict a priori, although a posteriori molecular dynamics and quantum chemical calculations combined with experimental 2D-NMR investigations have recently been reported for CDT-BTZ[89] (Figure 8). It was shown that there are several interchain packing geometries with different relative shifts of adjacent chains along the backbone direction, which differ only slightly in terms of their total energy. By comparing the 2D-NMR spectra and GIXRD data with the simulations, it was possible to identify the dominant packing geometry in the films. Fortunately, this geometry exhibits the largest transfer integrals and leads to the exceptionally high mobility values. However, this appears to be largely accidental, and it certainly suggests that a careful evaluation of different side chains is necessary for performance optimization in these systems.[65] There appears to be evidence from a comparison of many DPP materials[24] for a trend that side chains attached to the acceptor unit in addition to the (typically branched) solubilising side chains on the DPP donor unit tend to be detrimental or need at least to be positioned very carefully in order not to lead to reduced carrier mobility.[86,97] Very high performance with average hole and electron mobilities of 3.48 cm^2^ V^–1^ s^–1^ and 0.97 cm^2^ V^–1^ s^–1^, respectively, extracted from near ideal transfer characteristics was obtained for a selenophene-based DPP copolymer with a branched siloxane-based side in which the branching point is separated away from the backbone by an alkyl chain.[96]

**Figure 9:**
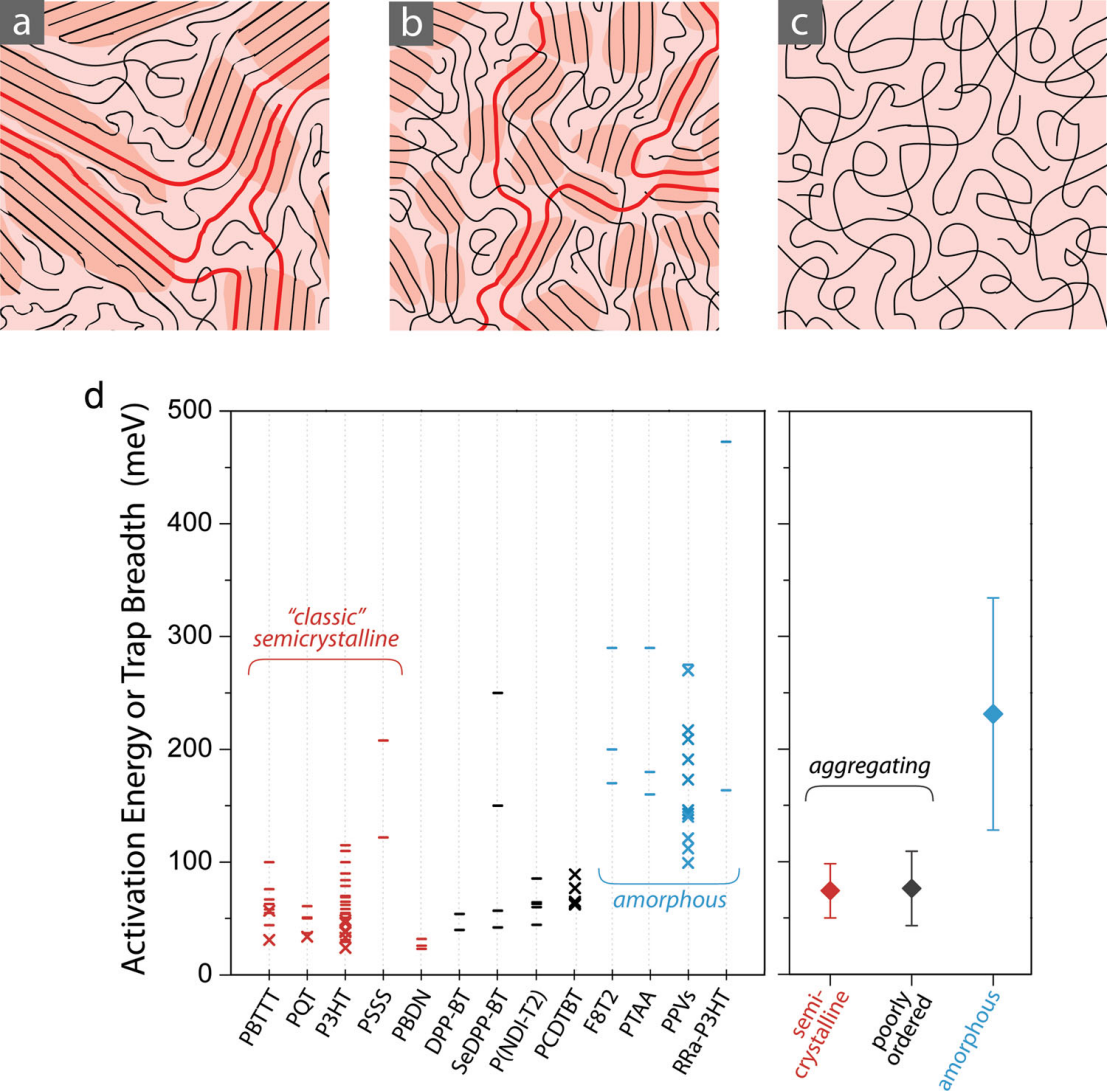
Microstructure of conjugated polymer films: a) semicrystalline polymer film, for example, P3HT; b) disordered aggregates typically of many donor-acceptor copolymers; c) completely amorphous film. There is a coexistence of ordered (darker shadowed areas) and spaghetti-like amorphous regions. Tie chains between aggregates are shown in bold red; d) activation energy for transport in semiconducting polymers; the plot includes FET data from the literature (dash), as well as trap depth/tail widths derived from device modelling (cross) for traditional classic semicrystalline materials (red), new high-performance polymers that are found to be poorly ordered (black), and completely amorphous materials (blue). Reproduced with permission.[98] _Copyright 2013, Macmillan Publishers Ltd._

In terms of a broader understanding of the underlying microscopic transport physics, Noriega et al.[98] recently proposed a classification of conjugated polymers into three categories based on their microstructure and degree of paracrystalline disorder (**Figure** 9). The paracrystallinity parameter *g* is defined as the standard deviation of local, static (cumulative) lattice fluctuations normalized by the average value of the lattice spacing. Values of *g* < 1% are typical of highly crystalline materials, whereas values of *g* > 10–20 % are characteristic of amorphous materials.[99,100]

- Semicrystalline conjugated polymers, such as P3HT and PBTTT, comprise a significant volume fraction of extended, crystalline aggregates, often with a lamellar packing, that give rise to well-defined diffraction spots, including higher order diffractions, in GIXRD studies. They exhibit relatively high field-effect mobilities (0.1–1 cm^2^ V^–1^ s^–1^). The presence of disordered, amorphous regions in the films does not necessarily hinder charge transport, as long as the molecular weight is high enough that there remains a percolating network of tie chains that provide interconnection between the aggregates. The residual energetic disorder encountered by charges is attributed to significant paracrystalline disorder in the π−π stacking distances within the aggregates (*g* ≈ 6–8%)[101] and manifests itself in moderate activation energies of the mobility or energetic width of trap distributions (*E*_a_ = 72 ± 24 meV).
- Poorly ordered but aggregating polymers adopt a more disordered microstructure with fewer observable and wider diffraction spots and larger paracrystallinity (*g* ≈ 10–15%) than in semicrystalline polymers, but they still contain small aggregates distinctly observable in GIXRD. Many of the recently discovered high mobility D–A copolymers discussed above appear to fall into this class. It is argued that analogous to semicrystalline polymers their high charge carrier mobility and comparable activation energies or trap depths (*E*_a_ = 76 ± 23 meV) are a reflection of the relatively unhindered motion of charge carriers between aggregates along tie chains and a relatively efficient interchain charge transfer within aggregates. In this percolation picture, charge carrier transport would then be relatively insensitive to the microstructure in the amorphous regions of the polymer film. The fact that some of these polymers exhibit mobilities significantly higher than semicrystalline polymers may be explained by the relatively large size of the conjugated molecular repeat units and close π−π stacking distances of these polymers, which may make interchain charge transfer integrals less sensitive to paracrystalline disorder in the π−π stacking within the aggregates.[98]
- Amorphous polymers, such as PTAA, adopt a highly disordered microstructure, in which scattering experiments, such as GIXRD, detect no evidence for aggregate formation. These polymers exhibit comparatively low mobilities < 0.1 cm^2^ V^–1^ s^–1^ and high activation energies *E*_a_ = 230 ± 100 meV.

**Figure 10:**
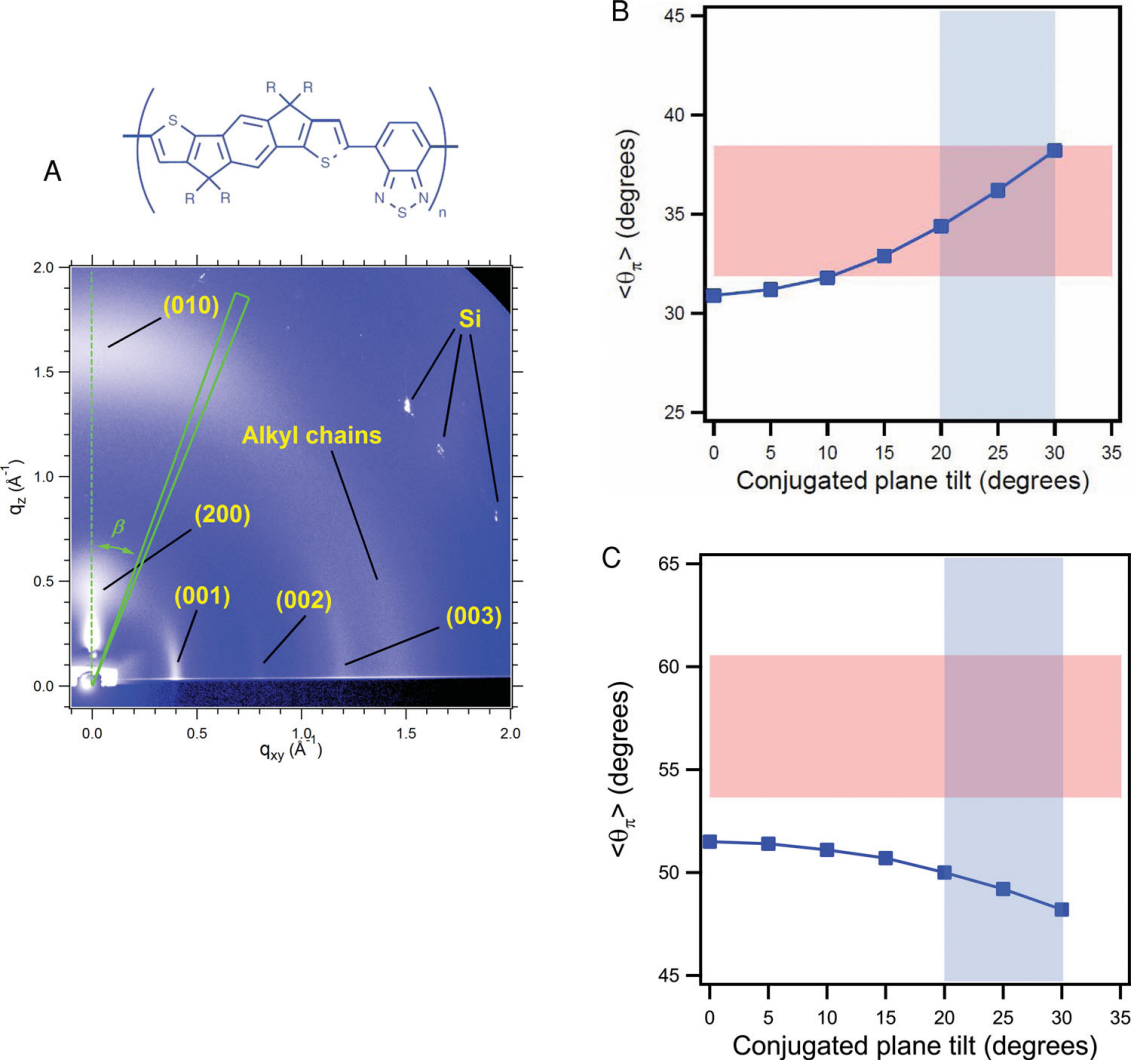
A) Wide angle X-ray scattering image of C_16_IDT-BT. B,C) Comparison of polymer chain orientation from GIXD and NEXAFS. The average orientation of the conjugated plane calculated from GIXD (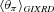) as a function of the conjugated plane tilt, i.e., the angle between the (010) reflection and the conjugated plane normal for C_16_IDT-BT (B) and fast-dried, non-annealed P3HT (C). The upper and lower bounds of the pink areas in a denote the average orientation of the conjugated plane that is compatible with NEXAFS spectra (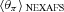) of top and buried interfaces, respectively. The light-blue areas highlight the conjugated plane tilt range between 20° and 30°: that is, the most probable conjugated plane tilt range for alkylated polythiophenes. For C_16_IDT-BT, the overlap between 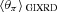 and the range of possible
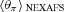
suggests that the conjugated planes are oriented in similar ways irrespective of whether their environments are crystalline or non-crystalline, whereas for P3HT the absence of overlap between the 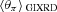 and 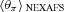 suggests that the orientation of the chains in non-crystalline environments is likely to be different from those in crystalline domains. Reproduced with permission.[81] Copyright 2013, Macmillan Publishers Ltd

A related, but somewhat nuanced microscopic explanation for the high mobilities (1–3 cm^2^ V^–1^ s^–1^) in IDT-BT was recently proposed by Zhang et al.[81] They identified an extended coplanarity of the backbone and an exceptionally uniform orientation of the conjugated molecular units with respect to the substrate plane not only in the crystalline, but also in the amorphous regions of the films in this polymer. The latter manifests itself in good agreement with the molecular orientation deduced from GIXRD (only sensitive to crystalline domains) and from NEXAFS (sensitive to all molecular units in the film) (**Figure** 10B), whereas in other polymers, there appears to be different orientations probed by GIXRD and NEXAFS (Figure 10C). In IDT-BT this allows for efficient fast intrachain transport along the extended, coplanar polymer backbone with occasional close, interchain contacts to enable efficient interchain hopping, which may not only be found in the aggregates but also in the more amorphous regions that lack sufficient long range order to manifest themselves in GIXRD.

More scientific work is clearly needed, and a full understanding of the microscopic nature of charge transport in these structurally rather complex, high-mobility polymers is not yet available at present. It is needed to provide clearer design guidelines for further materials improvement, for which there seems to be clear scope.

## 6. Outlook

In this review, we have discussed recent advances in the field of OFETs which have demonstrated that field-effect mobilities beyond those of amorphous silicon and in some cases approaching and even exceeding 10 cm^2^ V^–1^ s^–1^ are achievable in thin films of these weakly van der Waals bonded flexible, low-temperature, and solution-processable materials. These high values have been unexpected, certainly for conjugated polymer systems, and more work is clearly needed to improve the microscopic understanding of their complex microstructure, structure–property relationships and the origin of these high carrier mobilities. There appears to be clear scope for further improvement in materials performance, which should be guided by a much better microscopic understanding of their charge transport physics and control over the solution assembly to minimize structural and electronic defects. Achievement of mobilities of 1–10 cm^2^ V^–1^ s^–1^ in a relatively poorly ordered conjugated polymer challenge the applicability of traditional models of hopping transport in a disorder broadened density of states.[102] They raise the prospect of being able to enter into a transport regime that is no longer dominated by energetic disorder, but by the more intrinsic properties, polaronic properties of the charge carriers. This should allow observation of many fascinating, unique transport phenomena that have hitherto not been accessible in these systems.

As mobilities are approaching the requirements for demanding applications such as OLED active matrix addressing, an important, specific need arises to evaluate the constant current stress stability of these high mobility organic semiconductors under realistic operating conditions. There is already clear evidence that OTFTs can meet realistic lifetime requirements of several years of product use for voltage-driven displays, such as e-paper. However, whether the more demanding stability requirements for current-driven displays can be met needs now to be carefully evaluated. A scientific understanding is emerging of some of the instability mechanisms that give rise to operational threshold voltage shifts in OFETs,[20,21] but this needs to be extended to more representative device architectures, the new high-performance materials and applied to the inert environmental conditions that are typical in OLED packages.

In this review, I have not had space to discuss the scope of further improving the current drive capability and switching speed of OFETs through advanced, non-TFT device architectures. Over the past 10 years, a number of such architectures have been proposed and demonstrated. This includes vertical OFETs in which a short channel length is defined not by a lateral patterning step, but by the thickness of a film,[103–108] various metal-base transistor and grid triode architectures that could be directly integrated into an OLED stack[109–112] or a vertical diode design in which the bottom electrode is not continuous, but allows the electric field from an underlying gate electrode to penetrate into the active layer to modulate the charge injection from the source into the active layer.[113–118] There are difficult manufacturing challenges with most of these approaches, but if these could be solved the compatibility of organic semiconductors with such advanced architectures could potentially provide an additional boost to device performance over what further improvements in materials performance will deliver over the next couple of years.

The path of performance enhancement in organic semiconductors over the past 25 years is an inspiring example of the power of an interdisciplinary materials research effort bringing together organic chemists, materials scientists, device engineers, and physicists. Leading materials journals such as *Advanced Materials* have provided the community with very effective, rapid, and interdisciplinary dissemination channels and have made important contributions to the impressive progress made. Compared to important inorganic semiconductors, where key materials breakthroughs were often followed by focused and rapid materials optimization around relatively narrow materials compositions, the progress in organic semiconductors may have been more gradual, possibly because the incredible richness of mole­cular structures that can be synthesized by organic chemistry, and because the complex microstructure and structure–property relationships have made it more difficult to identify optimum molecular structures. However, there is now a powerful set of molecular modeling techniques,[119,120] microstructural probes[121] as well as spectroscopic and transport characterization techniques[5] that are increasingly making the materials discovery process less heuristic. As outlined in this review, there remain many fundamental research challenges that need to be addressed and surprises that are to be expected, but there is little evidence that the progress in materials performance has reached fundamental limits. It appears likely that organic field-effect transistors will over the next few years advance to performance levels that will allow these uniquely flexible and low-temperature, solution-processible materials to be used in a wide range of flexible electronic products with demanding performance requirements, including light-weight, unbreakable, flexible OLED displays on plastic substrates.
